# Electrostatically actuated encased cantilevers

**DOI:** 10.3762/bjnano.9.130

**Published:** 2018-05-08

**Authors:** Benoit X E Desbiolles, Gabriela Furlan, Adam M Schwartzberg, Paul D Ashby, Dominik Ziegler

**Affiliations:** 1 Molecular Foundry, Lawrence Berkeley National Laboratory, 1 Cyclotron Rd, Berkeley, California, USA; 2 Scuba Probe Technologies LLC, 255 Lina Ave, Alameda, California, USA

**Keywords:** amplitude calibration, atomic force microscopy, electrostatic excitation, encased cantilevers, liquid AFM

## Abstract

**Background:** Encased cantilevers are novel force sensors that overcome major limitations of liquid scanning probe microscopy. By trapping air inside an encasement around the cantilever, they provide low damping and maintain high resonance frequencies for exquisitely low tip–sample interaction forces even when immersed in a viscous fluid. Quantitative measurements of stiffness, energy dissipation and tip–sample interactions using dynamic force sensors remain challenging due to spurious resonances of the system.

**Results:** We demonstrate for the first time electrostatic actuation with a built-in electrode. Solely actuating the cantilever results in a frequency response free of spurious peaks. We analyze static, harmonic, and sub-harmonic actuation modes. Sub-harmonic mode results in stable amplitudes unaffected by potential offsets or fluctuations of the electrical surface potential. We present a simple plate capacitor model to describe the electrostatic actuation. The predicted deflection and amplitudes match experimental results within a few percent. Consequently, target amplitudes can be set by the drive voltage without requiring calibration of optical lever sensitivity. Furthermore, the excitation bandwidth outperforms most other excitation methods.

**Conclusion:** Compatible with any instrument using optical beam deflection detection electrostatic actuation in encased cantilevers combines ultra-low force noise with clean and stable excitation well-suited for quantitative measurements in liquid, compatible with air, or vacuum environments.

## Introduction

Dynamic atomic force microscopy requires excitation of the cantilever oscillation. Most commonly, this is achieved using a dither piezo built into the cantilever holder of the microscope. This technique is simple and effective, but it typically results in spurious peaks in the frequency response spectrum that are not directly related to the cantilever resonance. The origin of this so-called “forest of peaks” is attributed to mechanical resonances of the chip, chip holder, or dither piezo [1] along with modes of fluid vibration when working in liquids [2]. The problem is accentuated at high frequencies when operating in high-viscosity liquids. A user can easily select the wrong peak resulting in increased tip–sample interaction forces or unstable imaging conditions. More importantly, the indirect excitation of the cantilever is often very sensitive to changes to external factors causing drift over time of the excitation signal complicating imaging and preventing quantitative interpretation of image data [3–4]. Attempts to reduce spurious peaks include adding damping elements [5–6] or using alternative excitation methods such as resistive thermal [7–8], piezoelectric [9], electrostriction [10], or quartz-crystal tuning forks [11–12] that all solely excite the cantilever without inducing motion of the entire chip or the surrounding fluid. Magnetic excitation [13–15] or photothermal excitation [16–19] are more common in liquids, but require specialized instruments. Although frequently applied in microelectromechanical systems, electrostatic actuation is rarely used in scanning probe force microscopy. Reliable implementation remains difficult because alignment of an electrode is generally cumbersome and electrostatic forces frequently convoluted with the tip–sample interaction where changes in capacitance gradient due to topographical features influence cantilever excitation. An optically transparent electrode [20] or conductive sample [21–22] has been used as driving electrodes and Long et al. presented a designated cantilever holder to position an excitation electrode within few tens of micrometers on top of a regular cantilever [23]. However, accurate positioning in the confined space without perturbing the laser beam path remains very challenging. If the excitation electrode is approached too closely to the resonator, squeeze film damping or snap-in of the cantilever become another concern [24]. In our implementation with encased cantilevers these issues are solved by integrating the excitation electrode into the device itself. Built into the encasement and located between cantilever and sample, the actuation electrode does not need alignment and the device remains compatible with any scanning probe force microscope using optical beam deflection detection. Our method allows for static or dynamic actuation and is compatible with every scanning probe force microscope operating in vacuum, air, or liquid environments.

## Experimental

### Device concept

**Figure 1:**
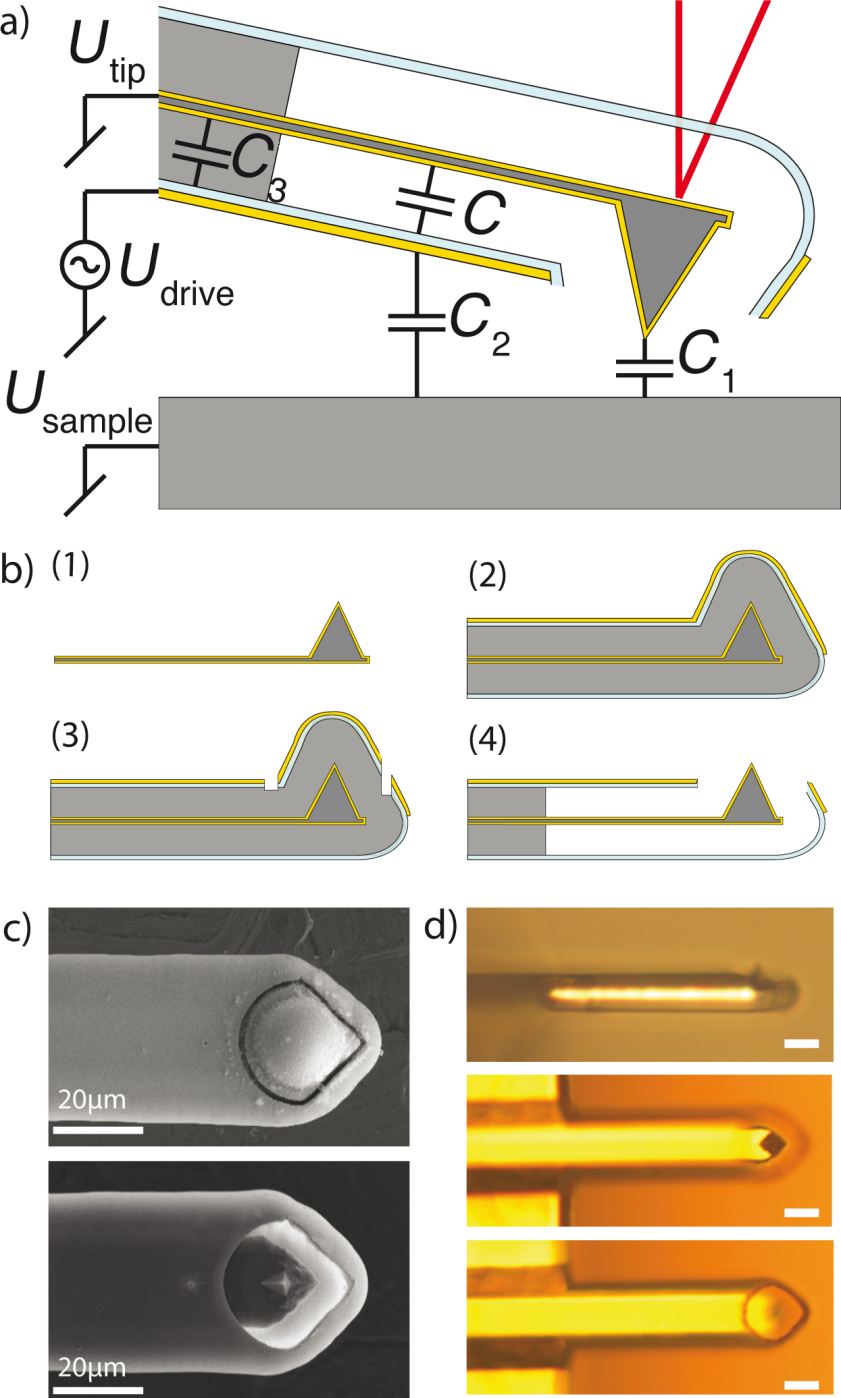
a) Cross section of an encased cantilever for electrostatic excitation. The electrical wiring and the capacitances involved are shown. The capacitance *C* is used for the actuation and the influence of parasitic capacitance (*C*_1_, *C*_2_, and *C*_3_) is discussed in greater detail in the Discussion section below. Integrating the excitation electrode at the bottom side of the encasement maintains a clear optical path for the laser based detection (shown in red). b) Simplified fabrication scheme with four process steps (1) gold-coated silicon cantilever, (2) deposition of sacrificial layer and encasement, (3) laser opening of encasement, and (4) release of cantilever by etchings sacrificial layer. c) Scanning electron microscopy image of the tip area before and after the release of the sacrificial layer. d) Optical images showing final devices at different angles and foci (scale bars = 20 μm).

Figure 1a shows a cross section of an encased cantilever with an integrated excitation electrode. The external electrical circuits show that the drive voltage (*U*_drive_) is applied to the built-in drive electrode. The electrical potential difference *U* between drive electrode and cantilever results in an attractive electrostatic force *F*_el_ = (1/2)*C*′*U*^2^, where *C*′ is the capacitance gradient. The tip–sample interactions remain unaltered by electrostatic forces of the integrated electrode, as both the cantilever (*U*_tip_) and sample (*U*_sample_) share a connection to a common ground at all times. The influence of parasitic capacitances (*C*_1_, *C*_2_, and *C*_3_) is discussed in greater detail in the Discussion section below.

### Device fabrication

Device fabrication is illustrated in Figure 1b. Beginning with gold-coated silicon cantilevers (NSC 19, Mikromasch) (1), we use chemical vapor deposition to coat a 11 μm thick sacrificial polymer layer followed by a 2 μm thick parylene layer. The first layer serves as sacrificial film that defines the air gap and the parylene builds the encasement. Next, we evaporate a 30 nm thick gold layer (2) that is the excitation electrode. Using a femtosecond pulsed laser we ablate a ca. 1 μm wide opening (3) to access the sacrificial methacrylate layer and etch it using organic solvents (1:3, methyl isobutyl ketone/isopropyl alcohol) during the subsequent release step (4). Figure 1c shows scanning electron microscopy images after step (3) and after the release of the sacrificial layer (4). During this wet-etch process the encasement over the probe apex falls off, leaving only a few micrometers of the sharp apex protruding from the encasement (see the optical images in Figure 1d).

### Experimental setup

For all experiments presented here we use an MFP-3D scanning probe force microscope (Asylum Research), with a liquid cell and the standard cantilever holder with a built-in actuation piezo. Only a slight modification of the standard holder is required to establish the two separate electrical contacts required for electrostatic actuation. To make an electrical connection to the tip we removed the polymer layers on the back side of the chip and inserted a thin sheet of gold foil between the chip and the cantilever holder. By contacting the gold foil and the metallic spring clip of the holder, we can provide reliable electrical contacts to the cantilever (*U*_tip_) and excitation electrode on the encasement (*U*_drive_).

## Results

### Experimental results

#### Electrostatic vs piezoacoustic excitation

**Figure 2:**
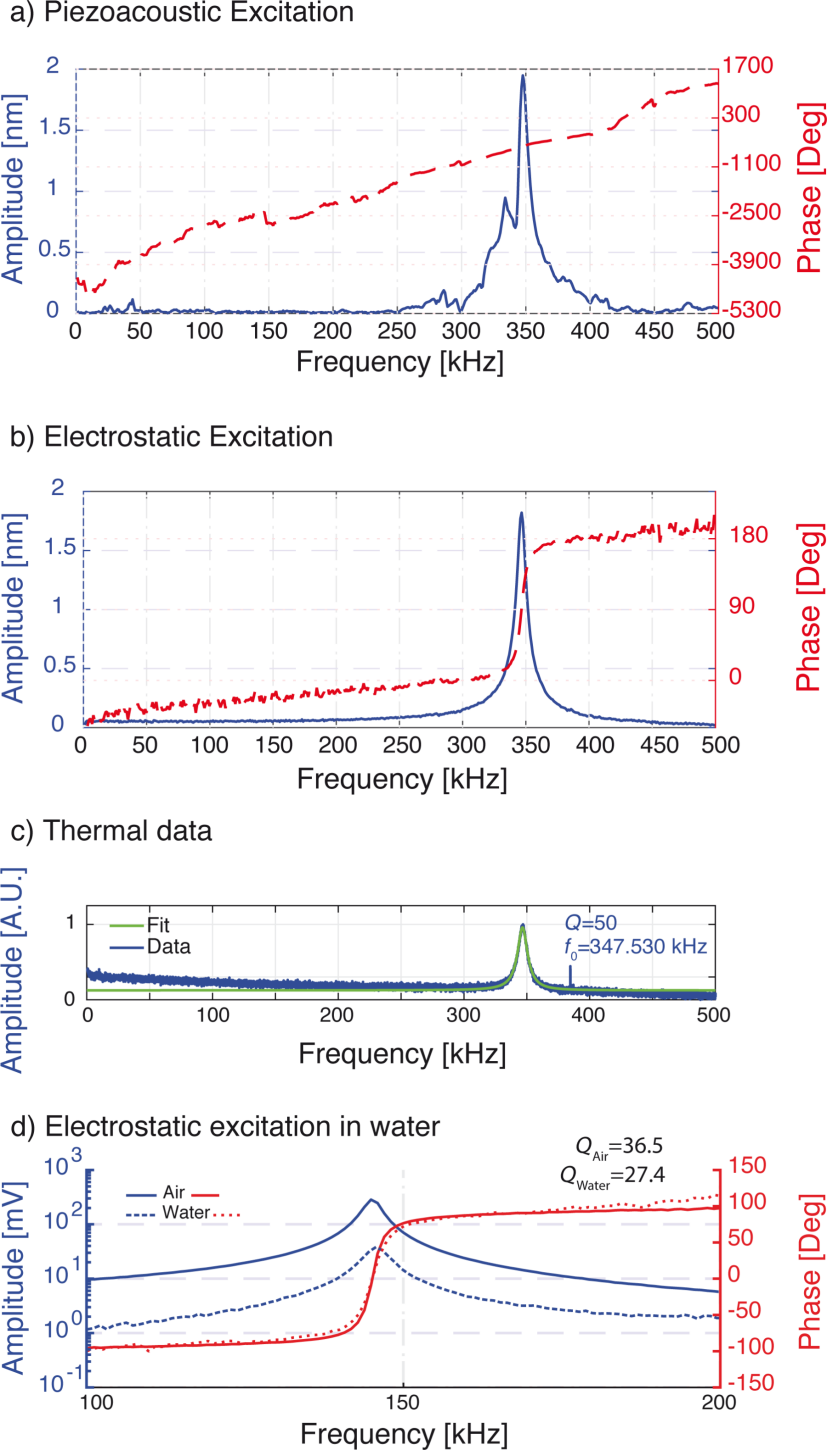
a) Amplitude (blue line) and phase (red dashed line) of the cantilever actuated using piezoacoustic excitation and b) electrostatic excitation. Piezoacoustic excitation shows spurious peaks through the entire spectrum while electrostatic excitation gives a clean resonance peak at *f*_0_ = 347.530 kHz, with a phase transition from 0° to 180°, and no spurious peaks over the entire frequency range (0 to 500 kHz). c) By fitting a simple harmonic oscillator model to the thermal power spectral density, one calculates resonance frequency, Q-factor and stiffness. d) Comparison of electrostatic excitation in air (continuous lines) and water (dotted lines). High frequency and Q-factors are maintained by air in the encasement.

Figure 2 compares piezoacoustic and electrostatic excitation in air. In both cases we record amplitude (blue line) and phase (red dashed line) while sweeping the excitation frequency from 0 to 500 kHz. Not uncommon for piezoacoustic excitation the resonance peak recorded in air shows extra features from the mechanical response of the cantilever holder (Figure 2a). However, electrostatic excitation (Figure 2b) results in a clean Lorentzian resonance peak with a smooth 180° phase transition. The resonance matches the thermal peak shown in Figure 2c. By fitting a harmonic oscillator model to the thermal peak (fit shown in green), we find a resonance frequency of *f*_0_ = 347.530 kHz, and a quality factor of *Q* = 50.0. Using the Sader method [25] we find a spring constant of *k*_dyn_ = 18 N·m^−1^. Figure 2d compares electrostatically excited resonance peaks for air and deionized water. For regular cantilevers without an encasement viscous losses to the surrounding medium are the dominate damping mechanism. The quality factor of the cantilever typically drops by a factor of 50 when immersed in water. By trapping air inside the hydrophobic encasement [26–29], the resonator maintains a high quality factor and resonance frequency. For the liquid air comparison a softer cantilever (*L* ≈ 90 μm) with a stiffness of *k* = 4 N·m^−1^ and a resonance frequency of *f*_0_ = 144.525 kHz and *Q*_air_ = 36.5 is used. After immersion in water the quality factor remained high *Q*_water_ = 27.4, which enables high-resolution imaging with small interaction forces in liquids. Moreover, the clean electrostatic excitation enables more reliable frequency modulation techniques [30] and generally results in quantitative measurements of tip–sample interactions.

#### Harmonic electrostatic excitation

In this section we analyze the harmonic excitation mechanism and the influence of *U*_drive_ in greater detail. The spectra shown in Figure 2b,c are recorded using harmonic excitation, where the excitation frequency ω_el_ matches the resonance frequency of the cantilever (ω_0_).

As introduced above, the electrostatic force is given by *F*_el_ = (1/2)*C*′*U*^2^. The electrical potential difference *U* is composed of *U*_drive_ and the contact potential difference *U*_CPD_ between cantilever and drive electrode. The drive voltage is *U*_drive_ = *U*_ac_ sin(ω_el_*t*) + *U*_dc_, where ω_el_ = 2π*f*_el_ is the angular frequency, *f*_el_ is the frequency and *t* is the time. The resulting electrostatic force *F*_el_ exhibits three spectral components as follows

[1]
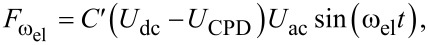


[2]
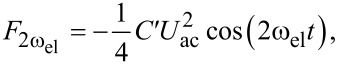


[3]
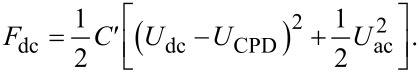


**Figure 3:**
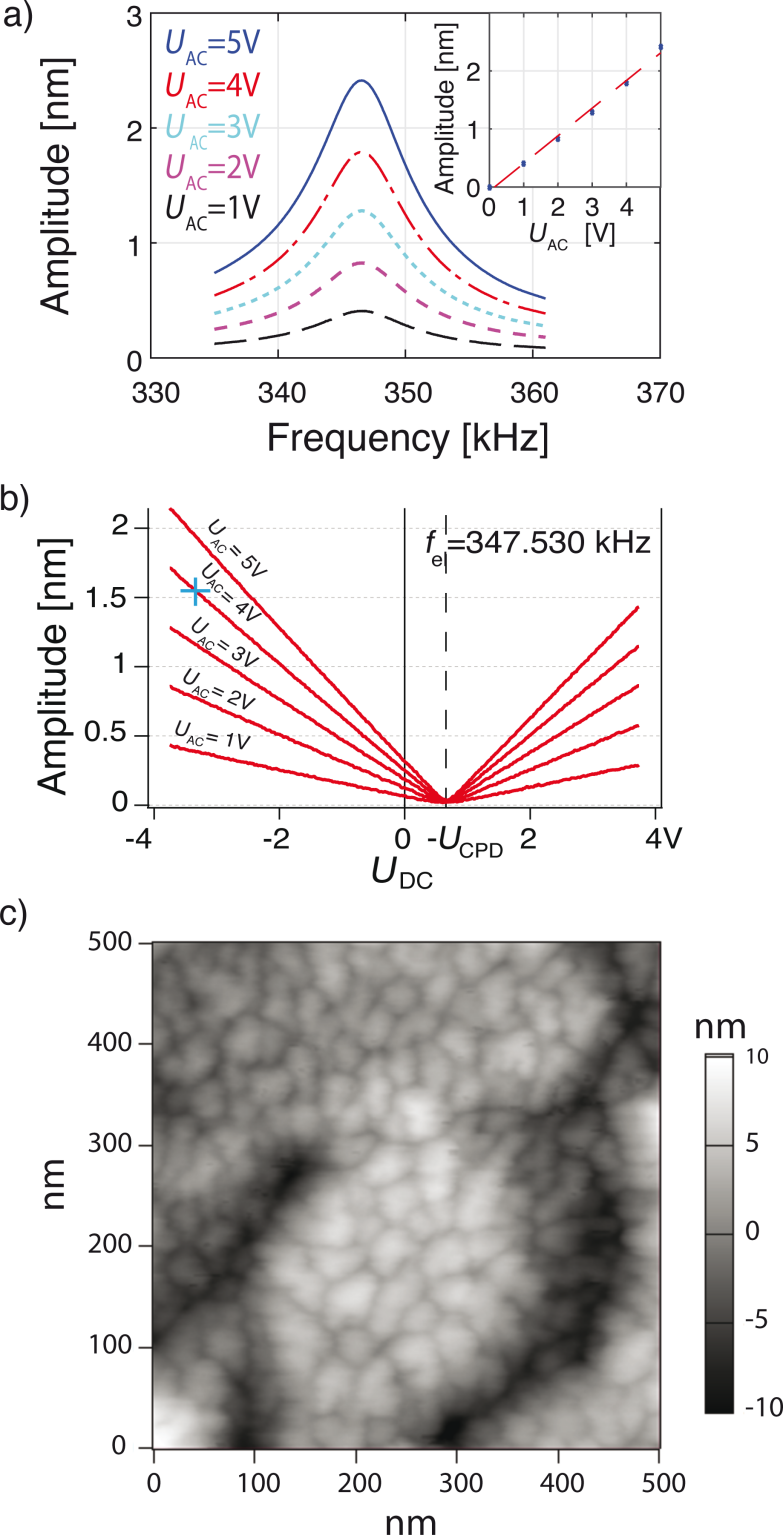
a) The amplitude increases linearly with the electrostatic drive signal (*U*_ac_), *U*_dc_ is held constant at −4 V. b) Amplitude as a function of *U*_dc_ plotted with *U*_ac_ ranging from 1 to 5 V. The amplitude increases linearly with |*U*_dc_ − *U*_CPD_|, and vanishes for *U*_dc_ = *U*_CPD_. The blue cross shows a single data point for |*U*_dc_ − *U*_CPD_| = *U*_ac_ = 4 V where we obtain an amplitude of 1.60 nm and it will be used for reference in the modeling section. c) Topographic image of copper grains evaporated onto an annealed ultra-flat gold surface. The image is recorded in air using electrostatic excitation with amplitude modulation feedback and a free amplitude of 1 nm and set-point of 0.8 nm.

Figure 3a shows the response of the cantilever at resonance (*f*_0_ = ω_0_/2π = 347.530 kHz). The amplitude increases linearly with *U*_ac_. *U*_ac_ is varied from 1 to 5 V in steps of 1 V and *U*_dc_ is maintained constant. Figure 3b shows the peak amplitude, *A*_ω_(ω_0_), as a function of *U*_dc_. Here, the applied bias *U*_dc_ is swept from −3.75 V to +3.75 V with *U*_ac_ varying from 1 to 5 V. As predicted by Equation 1, we observe a linear increase of the force and the resulting amplitude with *U*_dc_. Moreover, the amplitude vanishes for *U*_dc_ = *U*_CPD_ where 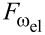 equals zero. Following the sign change of 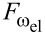 the phase shifts by 180° at this point (not shown). The experimentally determined amplitude can be expressed as 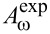 = 101 pm *V*^−2^|*U*_dc_ − *U*_CPD_|*U*_ac_. A comparison with modeled results is given in section Modeling.

We demonstrate imaging capability in air using electrostatic excitation in Figure 3c. A 3D rendering of the recorded topography shows copper grains evaporated onto an annealed ultra-flat gold surface. The surface is imaged in tapping mode using harmonic excitation with amplitude modulation feedback, a free amplitude of 1 nm and a set-point of 0.8 nm. In harmonic excitation, we observe that intentional switching of the applied *U*_dc_ by a few volts would result in a slow change of the amplitude with a time constant of several minutes before settling to a new steady state. This phenomenon can be explained by charging or polarizing of the parylene film altering the strength of the electrostatic excitation. Fabricating a gold layer on the inside of the encasement could potentially reduce this effect, but establishing reliable electrical contacts would be more difficult. As shown in the following section, this effect can be eliminated using sub-harmonic excitation, which is independent of static electric potentials.

#### Sub-harmonic electrostatic excitation

**Figure 4:**
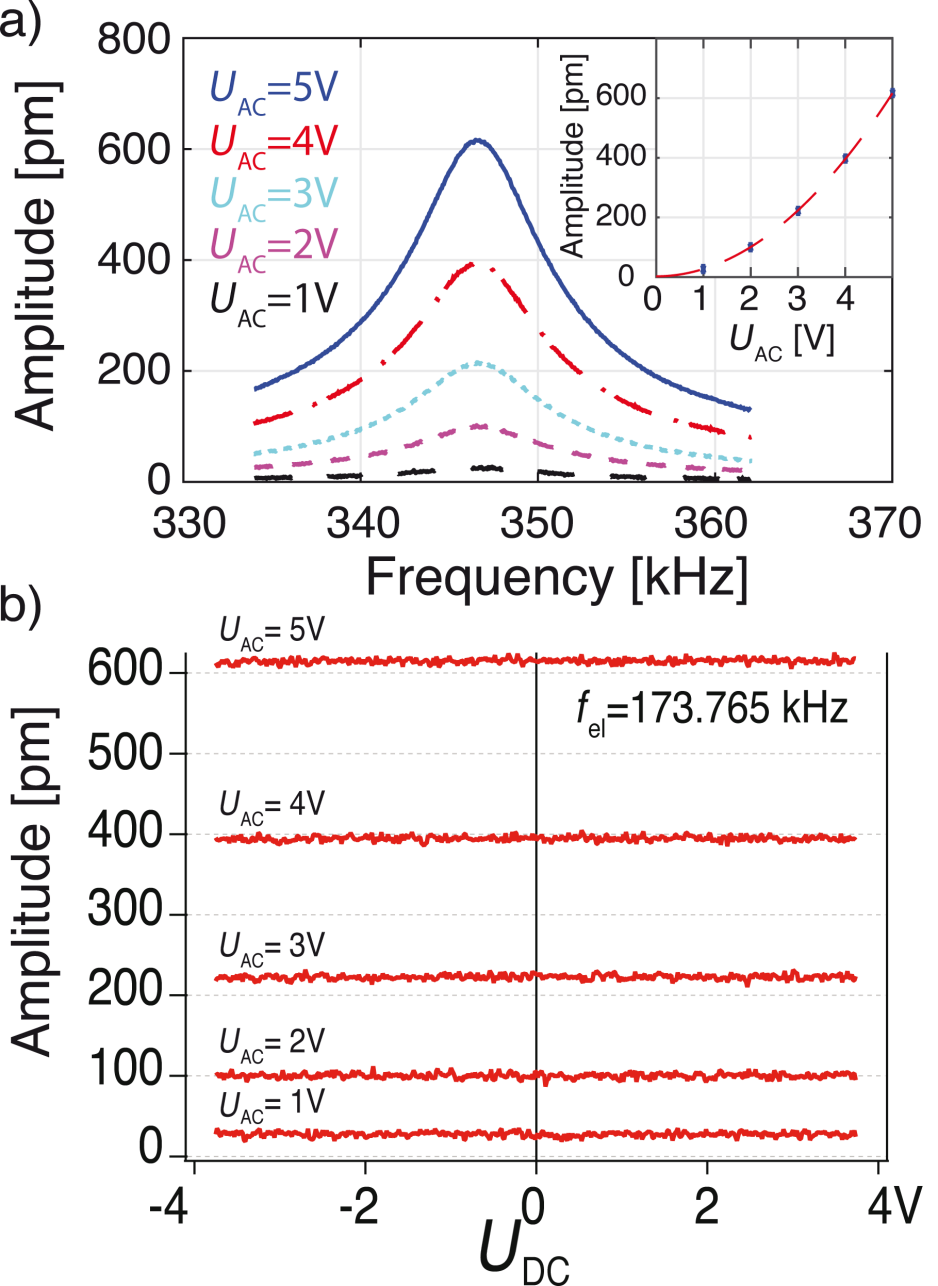
a) Oscillation amplitude at the resonance frequency of the cantilever induced by sub-harmonic excitation at half the resonance (*f*_el_ = *f*_0_/2 = 173.765 kHz). *U*_ac_ is varied from 1 V to 5 V and *U*_dc_ is constant at 5 V. The inset shows the quadratic increase in the peak amplitude with increasing *U*_ac_. b) The oscillation amplitude is independent on *U*_dc_.

In sub-harmonic excitation the drive frequency is set to half of the mechanical resonance (ω_el_ = ω_0_/2) such that the second-order force 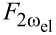 is actuating the cantilever at resonance (*f*_el_ = *f*_0_/2 = 173.765 kHz). Figure 4a shows the measured amplitude using sub-harmonic electrostatic excitation for constant *U*_dc_ = 5 V and *U*_ac_ varying from 1 to 5 V. As predicted, the electrostatic force and resulting amplitude increase quadratically with *U*_ac_. Figure 4b confirms that the oscillation amplitude is independent of *U*_dc_. Sweeping *U*_dc_ from −3.75 V to +3.75 V leaves the amplitude unaffected. Through fitting of our results we find that the resulting amplitude is best expressed by 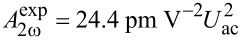. When compared to harmonic excitation, the same electrical potential results in a four-times smaller force (see Equations Equation 1 and Equation 2). For instance at |*U*_dc_ − *U*_CPD_| = *U*_ac_ = 4 V the oscillation amplitude induced by sub-harmonic excitation is approx. 400 pm, whereas it is approx. 1.60 nm using harmonic actuation (see blue cross in Figure 3b.).

#### Static cantilever deflection

**Figure 5:**
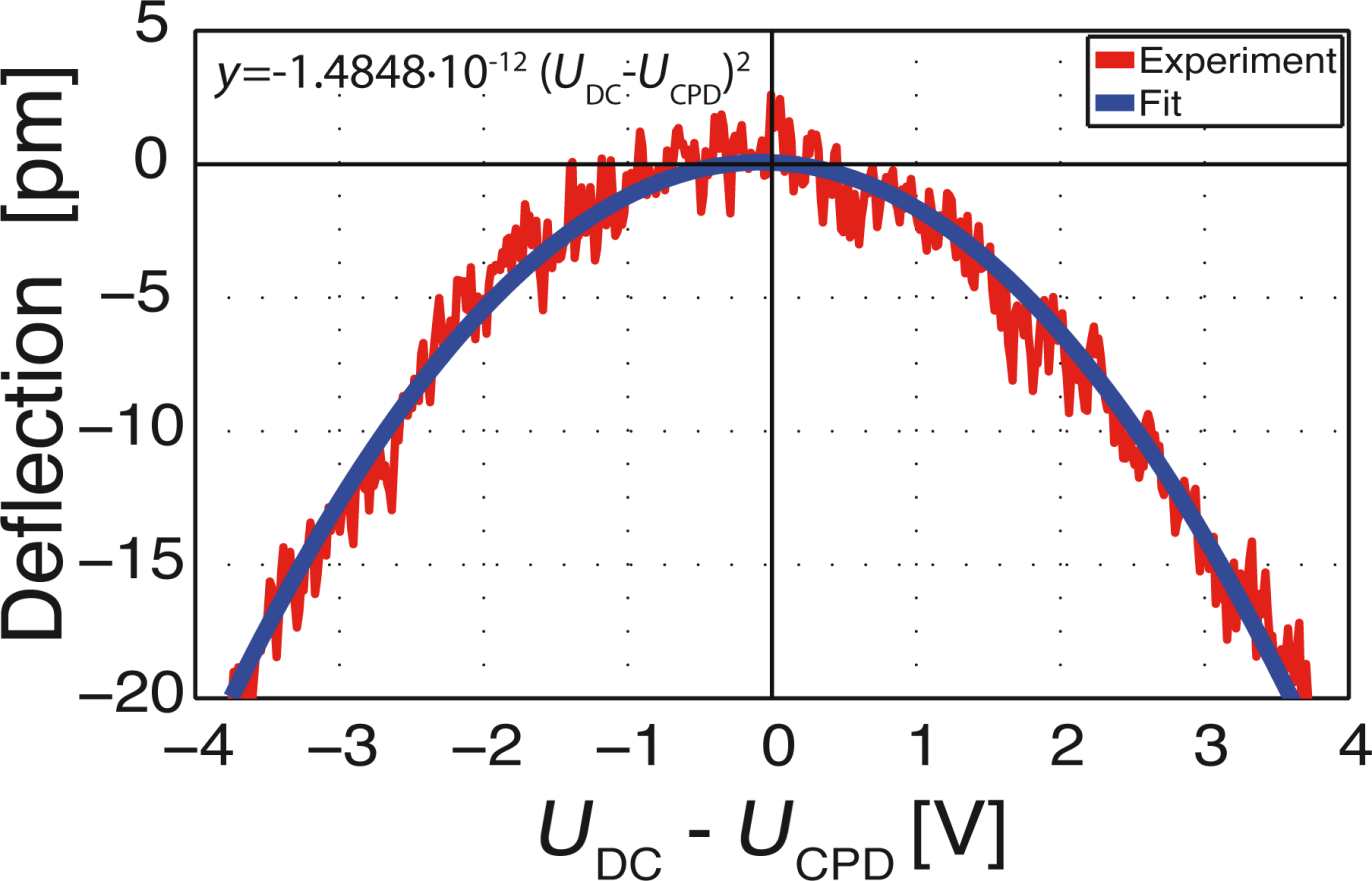
Measurement of the small downward deflection of the cantilever when varying the dc component (*U*_dc_ − *U*_CPD_). Negative deflection values represent bending towards the electrode. *U*_CPD_ is offset by ca. 0.65 V and *U*_ac_ = 0 V.

The effect of the static force *F*_dc_ (see Equation 3) remains to be observed. This force induces only small static deflection of a few picometers. The deflection is monitored as we sweep *U*_dc_ − *U*_CPD_ from −4 V to +4 V. (Figure 5). *U*_ac_ is maintained at zero and an offset of ca. 0.65 V is added to compensate *U*_CPD_. As expected from Equation 3, we observe a quadratic behaviour with *U*_dc_ − *U*_CPD_. Fitting a parabola (blue curve) we find that the experimentally found deflection is given by *y*^exp^ = −1.48 pm V^−2^ (*U*_dc_ − *U*_CPD_)^2^.

### Modeling

Despite the complex geometry of our drive electrode, we demonstrate that a simple parallel plate capacitor model can accurately describe the observed static deflection and oscillation amplitudes. After geometrical modeling of the capacitance gradient, we compute the expected deflection and amplitudes for static, harmonic and sub-harmonic excitation and compare them to our experimental results.

#### Geometric modeling of the capacitance gradient

**Figure 6:**
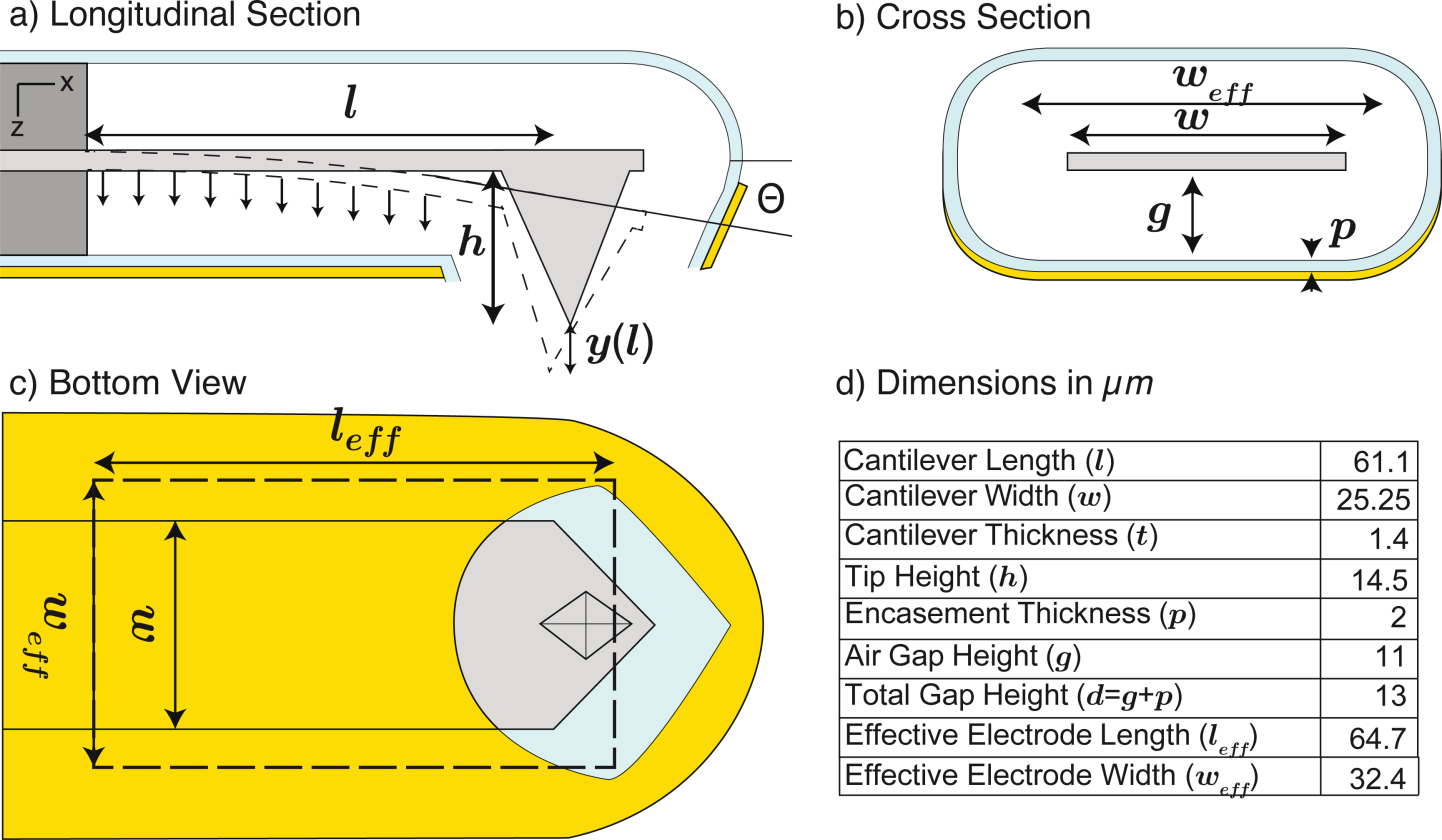
a) Longitudinal section of a cantilever with critical dimensions. The arrows illustrate the uniform loading induced by the electrostatic force resulting in a deflection (Θ) and displacement *y*(*l*) at the free end. b) Cross-sectional and c) bottom view showing effective electrode areas given by the product of *l*_eff_ and *w*_eff_. d) Table with all device dimensions in μm.

Figure 6 shows a schematic of the longitudinal (Figure 6a) and transverse (Figure 6b) cross section of the cantilever as well as a bottom view of the device (Figure 6c). The table in Figure 6d lists all of the dimensions of the device. Width (*w*) and thickness (*t*) of the cantilever, and height of the tip (*h*) are measured using scanning electron microscopy prior to the fabrication of the encasement. The length *l* of the free resonator, however, is set by the etching duration. Located inside the encasement its exact length is not easily measurable. Hence, we determine it from the resulting resonance frequency. The frequency of a tip-less beam in vacuum is well known and given by *f*_0_ = 0.162 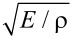 (*t*/*l*^2^), where *E* = 169 GPa is the Young’s modulus in the <110> direction of silicon [31] and ρ = 2330 kg·m^−3^ is the density of silicon. In our geometry, the tip significantly contributes to the total mass of the resonator. Therefore, a tip-mass-corrected frequency *f*_corr_ is applied [32].

[4]
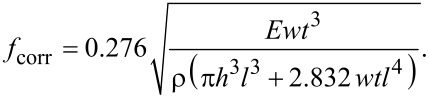


We solve for length with the known dimensions *h*, *t* and *w* and the measured resonance frequency of *f*_corr_ = 347.530 kHz and find *l* = 61.1 μm as the length of the cantilever.

In our geometry the gap (*g*) is comparable to the electrode width (*w*). Fringing field components must thus be taken into consideration [33–34] by modeling the apparently larger drive electrode with an effective width (*w*_eff_) and length (*l*_eff_) [35].

[5]
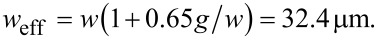


For the effective electrode length we include fringing field components only at the distal end of the cantilever:

[6]
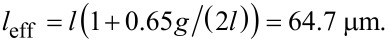


*A*_eff_ is the effective electrode area given by the product of the effective length (*l*_eff_) and width (*w*_eff_) as indicated in Figure 6c. The capacitor *C* is modeled as parallel plates built by the air gap (*C*_air_ = ε_0_*A*_eff_/*g*) in series with the parylene encasement (*C*_pary_ = ε_0_ε_pary_*A*_eff_/*p*).

[7]
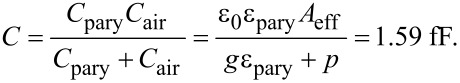


The resulting capacitance gradient *C*′ is

[8]
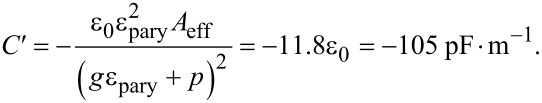


#### Static cantilever response

The capacitance gradient (*C*′) (see Equation 8) allows us to calculate the expected displacements for static and dynamic actuation. To this end, we assume a uniformly distributed load (*q*) given in force per unit length *q* = *F*_el_/*l* as indicated by the arrows in Figure 6a. Optical beam deflection measures the displacement of the tip *y*(*l*) indirectly by detecting the slope (Θ) at the laser position. After calibrating the optical lever sensitivity by recording a force–distance curve against a hard sample the displacement of the tip (*y*(*l*)) is known. The narrowing width at the end of the cantilever makes tip location and cantilever length (*l*) coincide. Hence, the well-known expression for deflection of a uniformly loaded cantilever can be used. *y*(*l*) = q*l*^4^/(8*EI*). Inserting the static stiffness *k*_stat_ = 3*EI*/*l*^3^ and *q* = *F*_dc_/*l* we can express *y*(*l*) as a function of the capacitance gradient (*C*′), stiffness (*k*_stat_) and electrical potentials (*U*_dc_, *U*_CPD_, and *U*_ac_):

[9]
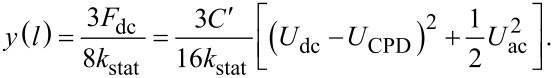


To compare this model with our experimental results we use the static stiffness *k*_stat_ = 12.9 N·m^−1^ and the modeled capacitance gradient *C*′ = −105 pF·m^−1^ to find 3 *C*′/16 *k*_stat_ = 1.51 pm·V^−2^. This value only differs by 2.4% from the experimentally found value of 1.48 pm·V^−2^ (see experimental results).

#### Harmonic cantilever response

For a dynamically actuated cantilever, the displacement is a function of the angular frequency (ω_el_) and reaches its maximum when ω_el_ coincides with the mechanical resonance frequency of the cantilever (ω_0_):

[10]
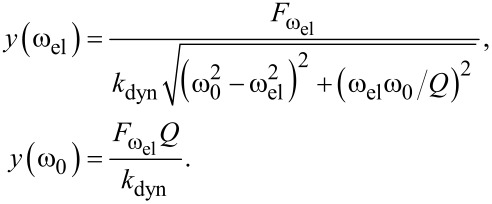


The amplitude for harmonic excitation at resonance is consequently given by

[11]
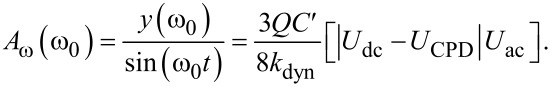


In contrast to static deflection, resonant excitation benefits from the mechanical gain resulting in an amplification by the quality factor (*Q*). We use the modeled capacitance gradient *C*′ = −105 pF·m^−1^, and the measured dynamic stiffness (*k*_dyn_ = 18.0 N·m^−1^) and *Q* = 50 found by the thermal method (see Figure 2c) to find a dynamic actuation factor of 109 pm·V^−2^. This model-based result is by 8% larger than the experimentally found value of 101 pm·V^−2^.

#### Sub-harmonic cantilever response

For modeling sub-harmonic excitation we simply substitute *F*_ω_ by *F*_2ω_ in Equation 10 to find a four-times smaller amplitude 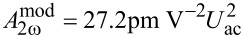. Table 1 below compares all model-based results with the experimentally found values.

**Table 1:** Comparison of the experimental and model-based results for amplitudes with harmonic and sub-harmonic actuation and static deflection.

	experiment	model	error

*A*_ω_	101 pm·V^−2^*U*_ac_|*U*_dc_ − *U*_CPD_|	109 pm·V^−2^*U*_ac_|*U*_dc_ − *U*_CPD_|	8%
*A*_2ω_	25.4 pm·V^−2^*U*_ac_^2^	27.2 pm·V^−2^*U*_ac_^2^	7.2%
*y*	−1.48 pm·V^−2^(*U*_dc_ − *U*_CPD_)^2^	−1.51 pm·V^−2^(*U*_dc_ − *U*_CPD_)^2^	2.4%

### Discussion

#### Calibration and validity of model

We find that our simple parallel-capacitor model overestimates static actuation by only a few percent. Considering the drastic simplifications of the electrode and tip geometry, as well as the general difficulty to measure stiffness of cantilevers accurately [36] the error of 2.4% is surprisingly small. Finding the length of the cantilever based on the measured resonance frequency was crucial to obtain accurate fits to theory. The larger, but still acceptable error for the dynamic modes (7.2% and 8%) can be explained by an underestimation of the dynamic stiffness by the Sader method [25,37]. The method leads to 
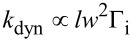
, where Γ_i_ is the imaginary part of the hydrodynamic function that depends on the Reynolds number, a function of the width of the cantilever, the frequency, fluid density and viscosity. However, Γ_i_ is unbounded, i.e., it does not include squeeze film damping effects [26]. Hence, it underestimates the stiffness, which results in a too large estimate for the amplitude.

Finite element analysis that takes into consideration squeeze film damping, as well as the complex shape of the drive electrode would be required to find the exact static and dynamic modal shapes. For a known geometry our model already predicts the amplitude well enough, such that a user can get a target amplitude by setting a drive voltage without requiring potentially tip-damaging calibration methods.

#### Excitation limits

Electrostatic actuation, especially when using sub-harmonic excitation gives extremely precise control over the oscillation amplitude. However, the largest achievable amplitude and static deflection are given by the maximum applicable voltage, which is limited by the breakthrough voltage of about 40 V of the sacrificial layer (see *C*_3_ in Figure 1a). For the used geometry this limits the maximum static deflection to about 200 pm. The pull-in deflection, where the electrostatic force exceeds the mechanical restoring force and the cantilever would snap in contact with the encasement represents the next limit. However, pull-in deflection is generally reached at one third of the gap, i.e., at 4 μm, which is orders of magnitude larger than the breakthrough-limited static deflection. With the relatively stiff cantilever used the attainable amplitude of a few nanometers might seem small. However, in absence of a water meniscus for example when operating in vacuum or when immersed in liquid, small amplitudes (below 1 nm) are ideal to reach gentle and high-resolution imaging conditions. Yet, larger amplitudes can be obtained with softer cantilevers. For instance with the cantilever that we characterized in Figure 2d (*k* ≈ 4 N·m^−1^) we achieved an amplitude of ca. 25 nm with drive voltages of 4 V.

#### Bandwidth limits

Capacitive excitation has a higher bandwidth than other cantilever excitation techniques. The parasitic capacitance spanning the tip-side of the entire silicon chip (*C*_3_) is ca. 100 pF, i.e., five orders of magnitudes larger than that of the excitation electrode (*C*). With a resistance of the electrical wiring to the cantilever of about 10 Ω such parasitic capacitance gives a bandwidth of 100 MHz. However, current limitations of amplifiers to drive this capacitive load lead to a practical bandwidth of ca. 5 MHz. Furthermore, cantilevers that oscillate at these frequencies are moderately stiff. We calculate that a cantilever with a stiffness of 20 N/m and resonance frequency of 5 MHz can be excited to an amplitude of 1 nm even with a moderate drive voltage of 5 V. Substantially stiffer cantilevers would have too small of an amplitude leading to a similar practical bandwidth of ca. 5 MHz. This is far greater than magnetic and piezo actuation techniques and similar to photothermal excitation.

#### Influence of the encasement

The capacitor between the encasement and the sample (See *C*_2_ in Figure 1a can build up an electrostatic force that actuates the encasement itself. As the top side of the encasement is transparent, such displacement would not be detected, nor would it actually influence the measurement done by the cantilever itself. Moreover, the structural stiffness of the encasement (*k*_enc_ = 757 N·m^−1^) exceeds the stiffness of the cantilever by far. Hence, possible electrostatically induced deflection of the excitation electrode (i.e., the encasement itself) can be neglected.

## Conclusion

We have demonstrated electrostatic actuation in encased cantilevers. We achieve clean resonance peaks in air and in liquid. The built-in drive electrode does not require any alignment. With only little modification of the cantilever holder, the devices can be used in almost any instrument. We studied static, harmonic and sub-harmonic actuation modes. In the harmonic mode, the frequency of the electrical drive signal matches the mechanical resonance frequency of the cantilever. In sub-harmonic mode, the excitation applies an ac drive voltage at half of the mechanical resonance frequency which results in more stable oscillation unaffected by fluctuations of polarization or charging states in the encasement material, the electrical contact potential or any other static offsets. Not requiring any dc voltage greatly reduces the risk of electrolytic production of gas bubbles. Electrostatic actuation in encased cantilevers provides more gentle imaging and more reliable interpretation of tip–sample interactions. The advantages over photothermal excitation are that no additional optical components or alignment procedures are required and that the cantilever does not get heated. With the used geometry and high stiffness, we achieve excitation of sub-nanometer amplitudes with high stability and extremely clean resonance behavior. Such small amplitudes greatly help reducing tip–sample interaction forces. Combining electrostatic actuation with encased cantilevers provides highly stable and low-noise force sensors that overcome the spurious mechanical resonances that are observed with traditional scanning probe methods under highly damped conditions in liquid.
